# Age at diagnosis in relation to survival following breast cancer: a cohort study

**DOI:** 10.1186/s12957-014-0429-x

**Published:** 2015-02-07

**Authors:** Jasmine Brandt, Jens Peter Garne, Ingrid Tengrup, Jonas Manjer

**Affiliations:** Department of Surgery, Lund University, Skåne University Hospital Malmö, Inga Marie Nilssons gata 47, SE-205 02 Malmö, Sweden; Department of Plastic Surgery, Lund University, Skåne University Hospital Malmö, Jan Waldenströmsgata 18, SE-205 02 Malmö, Sweden; Department of Breast Surgery, Aalborg Hospital, Aarhus University Hospital, Skovvej 3, 9000 Aalborg, Denmark

**Keywords:** Breast cancer, Survival, Age at diagnosis, Axillary lymph node status

## Abstract

**Background:**

Age is an important risk factor for breast cancer, but previous data has been contradictory on whether patient age at diagnosis is also related to breast cancer survival. The present study evaluates age at diagnosis as a prognostic factor for breast cancer on a large cohort of patients at a single institution.

**Methods:**

All 4,453 women diagnosed with breast cancer in Malmö University Hospital, Sweden between 1961 and 1991 were followed up on for 10 years with regards to breast cancer-specific mortality (BCSM) in different age groups. Corresponding relative risks (RR), with 95% confidence intervals, were obtained using Cox’s proportional hazards analysis. All analyses were adjusted for potential confounders and stratified for axillary lymph node involvement (ALNI) and diagnostic period.

**Results:**

As compared to women aged 40 to 49 years, those who were aged under 40 (RR: 1.40; 95% CI: 1.04 to 1.88) and 80 or more years (RR: 1.80; 95% CI: 1.45 to 2.25) had a statistically significant higher 10-year mortality rate. When adjusted for potential confounders, including stage at diagnosis, the associations only remained statistically significant for women aged 80 years or more. In the analyses stratified on ALNI, ALNI-negative women under 40 years had a statistically significant higher five-year mortality rate (RR: 2.65; 95% CI: 1.23 to 5.70). In the analyses stratified on diagnostic period, the positive association between women aged under 40 or aged 80 or more years and high BCSM rate remained, with statistically significant results for women aged 80 years or more in all periods.

**Conclusions:**

Women under 40 years of age had a poor prognosis, and this association was strongest among young women with axillary lymph node negative breast cancer. An age of 80 years or more was a prognostic factor for poor survival, independent of stage at diagnosis and diagnostic period.

## Background

Age is an important risk factor for breast cancer, but it has also been suggested that patient age at diagnosis is related to breast cancer survival [1,2]. It has been proposed that young and old age may be adverse prognostic factors, but data is conflicting [3-5]. As breast cancer is the most common malignancy in women under 40 years of age and approximately one third of breast cancer is diagnosed in women aged 70 years and older, it is important to clarify the association between age at diagnosis and breast cancer survival [2]. Previous data has been contradictory on whether the poor prognosis of young women exists in all stages, or only in women with small tumors and without axillary lymph node involvement (ALNI) [6-11]. Regarding the elderly, some studies have shown elderly women to have a poor outcome [5,12,13], whereas a few studies did not find an association [4,14] and one large study even found elderly women with ALNI-negative tumors to have a favorable outcome [15].

A potential reason as to why previous data has been conflicting may be that different cut-offs for age, as well as wide age groups have been used. Also, several studies have included only young or elderly women instead of all age categories. Furthermore, many previous studies have either consisted of small datasets or included several institutions. Follow-up time has in many studies also varied within the cohort, with the possible result that the patients with the longest follow-up may display spuriously low mortality rates, due to accumulated person-years.

The present cohort consists of a large unselected material of all women diagnosed with invasive breast cancer at a single institution in Sweden between 1961 and 1991. A total number of 4,453 cases were included and information was collected on clinical factors and tumor characteristics such as tumor size, ALNI, and distant metastasis [16]. The data has been followed up on through record-linkage with the Swedish Cause of Death Registry, and the entire cohort had the same follow-up time of 10 years. The large size of the cohort allowed the use of narrow age groups in the analyses, to study development over time, and stratification for other prognostic factors and diagnostic period.

The aim of the present study was to evaluate patient age at diagnosis as a prognostic factor for breast cancer on a large dataset at a single institution, and to examine the role of the factors included in stage at diagnosis (size, ALNI, and distant metastasis) and diagnostic period on this potential association.

## Methods

### The Malmö Breast Cancer Database

The study cohort consists of all cases of invasive female breast cancer in Malmö, Sweden, diagnosed between 1 January 1961 and 31 December 1991. They were all treated at the same institution, Malmö University Hospital, and no referrals were made to or from the hospital for patients with breast cancer. All residents in Sweden are registered by a unique 10-digit ID number. Breast cancer patients were identified by review of clinical notes and record-linkage with the Swedish Cancer Registry, forming the basis of the Malmö Breast Cancer Database. This was all completed by one surgeon, who also validated all breast cancer diagnoses by reviewing histological material, X-ray examinations, and medical records [16]. The present study was approved by the regional ethical committee in Lund, Sweden (approval number: LU-Dnr 615/2004).

### Clinical data and tumor characteristics

The surgeon identifying the cases and constructing the database also collected data regarding date of diagnosis, menopausal status, height, weight, parity, laterality, tumor location, and distant metastases through medical records and the Swedish Cancer Registry. Information concerning tumor size, histological type, and ALNI was retrieved from histopathological examinations. Tumor type was classified using a modification of the World Health Organization (WHO) classification as proposed by Linell *et al*. [17]. ALNI was divided into positive, negative, or unknown if no axillary dissection had been performed.

### Age at diagnosis

Age at diagnosis was obtained through a record-linkage between the Swedish Population Registry and the Swedish Cancer Registry. This information was available for all cases in the present study. The women were subsequently divided into six age groups: <40 years, 40 to 49 years, 50 to 59 years, 60 to 69 years, 70 to 79 years, and ≥80 years at the time of diagnosis.

### Follow-up

Follow-up was limited to 10 years following diagnosis. Two important reasons lay behind this decision; first, most recurrences and deaths from breast cancer occur within 10 years and second, patients diagnosed in the first diagnostic period accumulated a large number of person-years as compared to patients in later diagnostic periods. This would lead to spuriously low mortality rates for patients diagnosed during the first and second periods. To identify all deceased patients during the follow-up period, the cases’ ID-numbers were linked to the Swedish Cause of Death Registry (up until 31 December 2007), which contains information on date of death and underlying cause of death, as well as subordinate causes of death. The primary endpoint of this study was breast cancer as the underlying cause of death.

### Study population

A total of 4,453 women were diagnosed with breast cancer in Malmö during the study period (1961 to 1991). Out of these, 111 women were excluded as they obtained their breast cancer diagnosis at autopsy, 10 women were excluded due to missing information on all variables, 109 women were excluded because they had a previous diagnosis of breast cancer, and 104 women were excluded due to bilateral carcinomas. Consequently, the final study population consisted of 4,119 women.

### Statistical methods

The six age groups were compared concerning clinical factors and tumor characteristics. For the subsequent analyses the age categories were evaluated in relation to both a five-year and 10-year follow-up period. The time scale for the study was date of diagnosis until death or until end of the follow-up period, giving each individual a potential maximum of five and 10 years in the analyses, respectively. Missing values in covariates were coded as a separate category. Breast cancer-specific mortality (BCSM) rate was calculated per 10,000 person-years in different categories of age. Corresponding relative risks (RR) with 95% confidence intervals (CI) were obtained using Cox’s proportional hazards analysis. The proportional hazards assumption was confirmed using a log-minus-log plot. Women aged 40 to 49 years at diagnosis, were used as the reference. The reason for selecting this age category was that the youngest age group contained only 164 subjects, along with the fact that it has been suggested that the potential association between age and survival is bi-modal [13]. All Cox analyses were subsequently adjusted for factors making up tumor stage; tumor size, ALNI, and distant metastases. In a third model, all analyses were also adjusted for other potential prognostic factors. First, by adjusting for one factor at a time and secondly, for all of the potential prognostic factors simultaneously. The only variable not adjusted for was menopausal status, since nearly all women in the two youngest age groups were premenopausal, whereas all patients in the three oldest age groups were postmenopausal. Subsequently, all analyses were stratified for ALNI, as well as for diagnostic period, in order to reveal whether or not delayed diagnosis could have affected the results.

It has been suggested that women under 35 years of age may have a poor prognosis [18]. This was examined in a sensitivity analysis, subdividing women under 40 years of age into two groups; under 35 years and 35 to 39 years. This analysis used the same reference group (40 to 49 years).

## Results

### Patient characteristics with reference to age at diagnosis

Clinical factors and tumor characteristics of the six age groups are shown in Table 1. Distant metastasis at the time of diagnosis was more common with increasing age. There was considerably more missing data for the oldest age category (≥80 years) concerning axillary lymph node status. It was more common for older women to have been regarded as unsuitable for surgery, and it was also less common for these women to have undergone an axillary dissection.

**Table 1:** **Distribution of age at diagnosis in relation to patient characteristics, tumor characteristics, and surgical treatment**

**Factor**	**Age at diagnosis (years)**					
	**<40**	**40-49**	**50-59**	**60-69**	**70-79**	**≥80**
	**(n = 164)**	**(n = 630)**	**(n = 886)**	**(n = 1062)**	**(n = 818)**	**(n = 559)**
	Column percent^a^					
**Time of diagnosis**						
1961-1970	31	34	29	26	23	20
1971-1980	27	34	39	36	35	32
1981-1991	42	33	32	38	42	48
**Menopausal status**						
Premenopausal	97	90	21	0	0	0
Postmenopausal	3	10	79	100	100	100
**Body Mass Index**						
<20	16	11	6	4	5	6
20 - <25	48	43	37	28	26	17
25 - <30	9	16	20	26	26	17
≥30	2	4	8	11	10	5
Missing	25	26	29	31	33	55
**Parity (number of children)**						
Nullipara	23	20	21	25	28	28
1	26	25	27	25	22	16
2	32	32	31	25	19	12
3	14	15	12	12	13	7
≥4	3	3	5	7	9	10
Unknown	2	5	4	6	9	27
**Size of primary tumor (millimeters)**						
<5	2	2	4	2	2	1
5 - ≤10	9	12	16	15	10	7
10 - ≤20	30	29	30	34	33	25
20 - ≤50	31	25	23	26	31	33
>50	2	3	3	3	4	6
Missing	26	29	24	20	20	28
**Histological type**						
Tubular	4	8	8	6	8	4
Tubulo-ductal	9	13	14	18	17	15
Comedo	49	31	28	26	25	23
Lobular	4	10	8	9	9	8
Invasive, variable, unknown	14	12	12	11	11	22
Invasive, type not assessed	20	26	30	30	30	28
**Tumor location**						
Upper inner quadrant	19	15	18	18	18	16
Lower inner quadrant	7	8	7	7	7	6
Upper outer quadrant	42	43	43	42	41	38
Lower outer quadrant	12	13	11	13	12	11
Central	10	14	14	14	15	18
Missing	10	7	7	6	7	11
**Axillary lymph node status**						
Negative	51	50	49	55	46	22
Positive	46	44	42	36	34	22
Unknown	3	6	9	9	20	56
**Distant metastasis at diagnosis**						
No	99	97	95	94	91	90
Yes	1	3	5	6	9	10
**Type of surgery**						
Mastectomy	85	87	84	81	84	64
Partial mastectomy/local excision	15	13	14	16	9	14
Inoperable	0	0	0	3	7	22
**Axillary dissection**						
Yes	98	94	92	91	81	44
No	2	6	6	6	12	33
Inoperable	0	0	2	3	7	22

N = number of patients in each age category.

^a^ Due to some missing values, percentages do not always add to 100%.

### Age at diagnosis in relation to survival

Young age (<40 years) was positively associated with a high BCSM in both the five-year follow-up period and in the 10-year follow-up period, although it was only statistically significant in the 10-year follow-up period (Table 2). However, the association disappeared when adjusted for stage and other potential prognostic factors. The two oldest age categories, 70 to 79 and ≥80 years, had a statistically significant higher BCSM as compared to the reference group in both the five- and 10-year follow-up period. Women aged 80-years-old or more displayed the worst outcome, and this association remained statistically significant after adjustment for all potential confounders.

**Table 2:** **Age at diagnosis in relation to breast cancer-specific mortality**

	**Age at diagnosis (years)**	**Subjects**	**Person-years**	**Breast cancer deaths (all deaths)**	**Breast cancer mortality/10,000** ^**a**^	**Relative risk (95% CI)**	**Relative risk** ^**b**^ **(95% CI)**	**Relative risk** ^**c**^ **(95% CI)**
5-year follow-up	<40	164	722	40 (45)	554	1.34 (0.94-1.92)	0.95 (0.72-1.25)	0.85 (0.65-1.12)
	40-49	630	2,846	117 (133)	411	1.00	1.00	1.00
	50-59	886	3,801	195 (226)	513	1.25 (0.99-1.57)	1.08 (0.88-1.33)	1.07 (0.87-1.31)
	60-69	1,062	4,559	204 (283)	448	1.09 (0.87-1.36)	1.02 (0.83-1.26)	1.08 (0.87-1.34)
	70-79	818	3,146	177 (330)	563	1.36 (1.08-1.72)	1.02 (0.82-1.28)	1.15 (0.91-1.45)
	≥80	559	1,607	130 (378)	809	1.93 (1.50-2.48)	1.32 (0.96-1.83)	1.53 (1.08-2.16)
	All	4,119	16,681	863 (1395)	517			
10-year follow-up	<40	164	1,251	59 (66)	472	1.40 (1.04-1.88)	0.96 (0.77-1.20)	0.89 (0.71-1.12)
	40-49	630	5,173	171 (193)	331	1.00	1.00	1.00
	50-59	886	6,817	276 (337)	405	1.22 (1.01-1.47)	1.11 (0.94-1.32)	1.08 (0.91-1.28)
	60-69	1,062	8,017	283 (445)	353	1.05 (0.87-1.27)	1.06 (0.89-1.26)	1.08 (0.90-1.29)
	70-79	818	5,034	240 (543)	477	1.37 (1.12-1.66)	0.99 (0.82-1.21)	1.10 (0.90-1.35)
	≥80	559	2,168	147 (497)	678	1.80 (1.45-2.25)	1.44 (1.07-1.93)	1.61 (1.18-2.20)
	All	4,119	28,460	1,176 (2081)	413			

CI = Confidence Intervals.

^a^Breast cancer-specific mortality per 10,000 person-years.

^b^Adjusted for tumor size, axillary lymph node status, and distant metastasis.

^c^Adjusted for diagnostic period, BMI, parity, tumor size, axillary lymph node status, histological type, tumor location, and distant metastasis.

When adjusting for confounders one by one, the two factors that affected the results the strongest were ALNI and tumor size. ALNI decreased the youngest age category’s RR from 1.34 (95% CI: 0.94 to 1.92) to 0.97 (95% CI: 0.74 to 1.27) and the oldest age category’s RR from 1.93 (95% CI: 1.50 to 2.48) to 1.09 (95% CI: 0.78 to 1.51) in the five-year follow-up period.

In the sensitivity analysis, women under 35 years of age (53 women) had an RR of 1.36 (95% CI: 0.77 to 2.42) and women 35 to 39 years (111 women) had an RR of 1.36 (95% CI: 0.89 to 2.06). In the 10-year follow-up period, women aged 35 to 39 had a statistically significant RR of 1.50 (95% CI: 1.07 to 2.11), whereas the result for women under 35 did not reach statistical significance, with an RR of 1.23 (95% CI: 0.75 to 2.02).

### Age at diagnosis and survival in relation to axillary lymph node involvement

**Figure 1:**
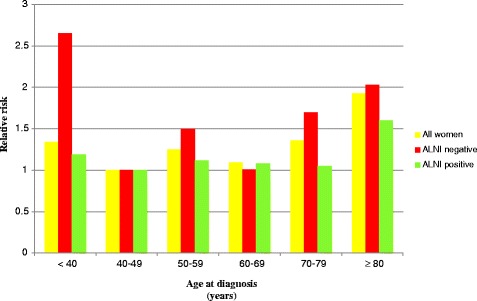
**Age at diagnosis in relation to breast cancer-specific mortality with stratification for axillary lymph node involvement (ALNI); five-year follow-up period.**

Young age (<40 years) was associated with a high BCSM among ALNI-negative women in the five- and 10-year follow-up periods (Table 3, Figure 1). The association was statistically significant in the five-year follow-up period with an RR of 2.65 (95% CI: 1.23 to 5.70). Ten-year BCSM rates were also high but were not statistically significant, with an RR of 1.69 (95% CI: 0.92 to 3.09). Following adjustments for all potential factors, the point estimate among ALNI-negative women followed up on for five years was still high, but it did not reach statistical significance.

**Table 3:** **Age at diagnosis in relation to breast cancer-specific mortality with stratification for axillary lymph node status**

**Axillary lymph node status**		**Age at diagnosis (years)**	**Subjects**	**Person-years**	**Breast cancer deaths (all deaths)**	**Breast cancer mortality/10,000** ^**a**^	**Relative risk (95% CI)**	**Relative risk** ^**b**^ **(95% CI)**	**Relative risk** ^**c**^ **(95% CI)**
Negative	5- year follow-up	<40	84	399	11 (12)	276	2.65 (1.23-5.70)	2.40 (1.11-5.17)	1.91 (0.88-4.19)
		40-49	313	1,531	16 (23)	105	1.00	1.00	1.00
		50-59	434	2,052	32 (48)	156	1.50 (0.82-2.73)	1.68 (0.92-3.06)	1.64 (0.90-3.02)
		60-69	579	2,761	29 (65)	105	1.01 (0.55-1.86)	0.92 (0.49-1.71)	0.87 (0.46-1.64)
		70-79	373	1,661	29 (81)	175	1.70 (0.92-3.12)	1.50 (0.81-2.80)	1.35 (0.71-2.58)
		≥80	123	488	10 (57)	205	2.03 (0.92-4.47)	1.69 (0.76-3.78)	1.88 (0.82-4.30)
		All	1,906	8,890	127 (286)	143			
	10-year follow-up	<40	84	741	15 (18)	202	1.69 (0.92-3.09)	1.53 (0.84-2.81)	1.29 (0.70-2.38)
		40-49	313	2,912	35 (47)	120	1.00	1.00	1.00
		50-59	434	3,881	58 (90)	149	1.25 (0.82-1.90)	1.38 (0.90-2.10)	1.44 (0.94-2.20)
		60-69	579	5,119	57 (143)	111	0.93 (0.61-1.41)	0.89 (0.58-1.36)	0.90 (0.58-1.38)
		70-79	373	2,860	60 (187)	210	1.75 (1.15-2.66)	1.55 (1.01-2.37)	1.51 (0.97-2.35)
		≥80	123	714	13 (98)	182	1.54 (0.81-2.91)	1.25 (0.66-2.38)	1.44 (0.74-2.78)
		All	1,906	16,228	238 (583)	147			
Positive	5- year follow-up	<40	75	301	28 (32)	930	1.19 (0.78-1.82)	1.33 (0.87-2.04)	1.07 (0.69-1.66)
		40-49	274	1,141	89 (95)	780	1.00	1.00	1.00
		50-59	376	1,498	130 (139)	868	1.12 (0.85-1.46)	1.23 (0.94-1.61)	1.29 (0.98-1.70)
		60-69	383	1,526	128 (158)	839	1.08 (0.82-1.41)	1.20 (0.92-1.58)	1.30 (0.98-1.72)
		70-79	283	1,059	86 (134)	812	1.05 (0.78-1.41)	1.11 (0.82-1.50)	1.21 (0.88-1.66)
		≥80	124	383	47 (81)	1,227	1.60 (1.13-2.29)	1.38 (0.96-1.98)	1.68 (1.14-2.25)
		All	1,515	5,908	508 (639)	860			
	10-year follow-up	<40	75	472	42 (46)	890	1.39 (0.98-2.00)	1.55 (1.09-2.21)	1.34 (0.94-1.93)
		40-49	274	1,954	121 (128)	619	1.00	1.00	1.00
		50-59	376	2,530	177 (198)	700	1.13 (0.90-1.42)	1.25 (0.99-1.57)	1.26 (1.00-1.60)
		60-69	383	2,461	174 (228)	707	1.12 (0.89-1.42)	1.25 (0.99-1.58)	1.27 (1.00-1.63)
		70-79	283	1,580	112 (217)	709	1.10 (0.85-1.42)	1.14 (0.88-1.48)	1.22 (0.93-1.60)
		≥80	124	523	53 (106)	1,013	1.54 (1.11-2.13)	1.30 (0.93-1.81)	1.61 (1.13-2.28)
		All	1,515	9,520	679 (923)	713			

CI = Confidence Intervals.

^a^ Breast cancer-specific mortality per 10,000 person-years.

^b^Adjusted for tumor size and distant metastasis.

^c^Adjusted for diagnostic period, BMI, parity, tumor size, histological type, tumor location, and distant metastasis.

Women aged 70 to 79 years who were ALNI-negative also displayed a high BCSM. The oldest age group (≥80 years) had a high BCSM among both ALNI-negative and ALNI-positive women throughout both the five- and 10-year follow-up periods.

In the sensitivity analysis, ALNI-negative women aged 35 to 39 years (111 women) displayed a statistically significant RR of 3.50 (95% CI: 1.59 to 7.70) and women under 35 years (53 women) an RR of 0.82 (95% CI: 0.11 to 6.17) in the five-year follow-up period. Results were similar in the 10-year follow-up period.

### Age at diagnosis and survival in relation to diagnostic period

In the results stratified for diagnostic period, young age (<40 years) continued to be related to poor prognosis throughout the diagnostic periods in the five-year follow-up, although the results were not statistically significant (Table 4). The oldest age category (≥80 years) displayed the highest BCSM in all diagnostic periods. The results were similar in the 10-year follow-up for all age categories.

**Table 4:** **Age at diagnosis in relation to breast cancer-specific mortality with stratification for diagnostic period; five-year follow-up period**

**Diagnostic period**	**Age at diagnosis (years)**	**Subjects**	**Person-years**	**Breast cancer deaths (all deaths)**	**Breast cancer mortality/10,000** ^**a**^	**Relative risk (95% CI)**	**Relative risk** ^**b**^ **(95% CI)**	**Relative risk** ^**c**^ **(95% CI)**
1961-1970	<40	51	214	16 (18)	747	1.39 (0.79-2.45)	1.69 (0.96-2.98)	1.23 (0.69-2.20)
	40-49	214	940	50 (56)	532	1.00	1.00	1.00
	50-59	256	1,008	82 (88)	813	1.53 (1.07-2.17)	1.51 (1.06-2.16)	1.46 (1.00-2.11)
	60-69	274	1,107	65 (93)	587	1.10 (0.76-1.59)	1.15 (0.79-1.67)	1.12 0-75-1.66)
	70-79	189	673	45 (92)	669	1.25 (0.83-1.86)	1.33 (0.88-2.02)	1.35 (0.87-2.09)
	≥80	108	252	29 (82)	1,151	2.10 (1.33-3.32)	1.39 (0.86-2.27)	1.21 (0.72-2.03)
	All	1,092	4,193	287 (429)	684			
1971-1980	<40	44	201	8 (10)	399	1.16 (0.54-2.51)	1.17 (0.54-2.55)	1.09 (0.50-2.39)
	40-49	211	960	33 (39)	344	1.00	1.00	1.00
	50-59	343	1,535	65 (76)	423	1.23 (0.81-1.87)	1.15 (0.75-1.77)	1.18 (0.76-1.82)
	60-69	386	1,633	84 (116)	514	1.49 (1.00-2.23)	1.65 (1.09-2.48)	1.69 (1.11-2.57)
	70-79	287	1,084	72 (122)	664	1.92 (1.27-2.90)	1.78 (1.16-2.72)	1.81 (1.17-2.81)
	≥80	181	518	44 (119)	849	2.43 (1.55-3.82)	2.27 (1.38-2.73)	2.41 (1.44-4.02)
	All	1,452	5,931	306 (482)	516			
1981-1991	<40	69	307	16 (17)	521	1.45 (0.80-2.62)	1.49 (0.82-2.70)	1.15 (0.62-2.13)
	40-49	205	946	34 (38)	359	1.00	1.00	1.00
	50-59	287	1,258	48 (62)	382	1.06 (0.68-1.65)	1.30 (0.84-2.03)	1.33 (0.85-2.09)
	60-69	402	1,819	55 (74)	302	0.84 (0.55-1.29)	0.85 (0.55-1.31)	0.90 (0.58-1.41)
	70-79	342	1,389	60 (116)	432	1.20 (0.79-1.82)	0.67 (0.43-1.04)	0.82 (0.52-1.30)
	≥80	270	837	57 (177)	681	1.88 (1.23-2.87)	0.79 (0.50-1.24)	1.06 (0.65-1.72)
	All	1,575	6,557	270 (484)	412			

CI = Confidence Intervals.

^a^Breast cancer-specific mortality per 10,000 person-years.

^b^Adjusted for tumor size, axillary lymph node status and distant metastasis.

^c^Adjusted for BMI, parity, tumor size, axillary lymph node status, histological type, tumor location, and distant metastasis.

**Figure 2:**
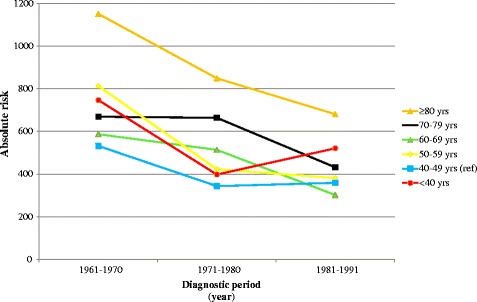
**Age at diagnosis in relation to breast cancer-specific mortality/10,000 person-years with stratification for diagnostic period; five-year follow-up period.**

Figure 2 displays the absolute risks, measured as BCSM per 10,000 person-years, which can also be found in Table 4. Mortality rates decreased for all age groups from the first to the last diagnostic period. The decrease was most apparent for women aged 50 to 59 and 60 to 69-years-old. Being diagnosed between 1981 and 1991 was associated with the best prognosis for all age categories, except for the youngest age category and the reference category, which instead had a lower BCSM in the diagnostic period 1971 to 1980. Women aged 80 years or more continued to show a significantly higher absolute risk compared to all other age categories throughout the diagnostic periods.

## Discussion

In this study, young (<40 years) and old (≥80 years) age was positively associated with a high BCSM. For women aged under 40 years, this was predominantly apparent for those with ALNI-negative breast cancer. An age of 80 years or more was a prognostic factor for high BCSM, independent of stage and diagnostic period.

### Strengths and limitations

This study was conducted among women diagnosed between 1961 and 1991 and due to this some information of interest unfortunately could not be collected at the time of diagnosis. However, to our knowledge this is one of the largest population-based patient datasets with all women treated at the same institution. It consisted of 4,453 women, with a 10-year follow-up period for the entire cohort. No referrals were made to or from the hospital for patients with breast cancer. All women diagnosed with invasive breast cancer in Malmö between 1961 and 1991 were included, minimizing a potential selection bias. Age at diagnosis and breast cancer diagnosis was obtained for all patients from the Swedish Population Registry and the Swedish Cancer Registry. Both these registries are highly valid [19,20]. Histopathological examination was performed on all samples at a single pathology department; hence the reliability of tumor characteristics ought to have been high [16]. Cause of death was obtained from the Swedish Cause of Death Registry. The validity of the Swedish Cause of Death Registry has been evaluated for cause of death amongst breast cancer patients diagnosed in Malmö in two studies and found to be highly accurate [16,21].

The most important data that we were not able to adjust for due to information not being collected were histological grade, hormone receptor status, and adjuvant treatment. Data on the type of operation and whether an axillary dissection was performed is displayed in Table 1. The results were stratified for diagnostic period, which may have decreased the risk of confounding caused by treatment. In all periods adjuvant therapy was in general given routinely according to factors such as age, and ALNI; factors that were included in the analysis. This may have adjusted for treatment to some extent. However, it is still likely that older women less frequently would have been given adjuvant treatment according to the guidelines, which would have led to a lower survival rate. This is discussed further in the section below on older women. Also, women under 40 years may have been given more aggressive treatment than the guidelines indicated. For young women, it may therefore be the opposite, that is, they may have been treated more actively using, for example, chemotherapy, and their survival rate may have been even lower if we had been able to adjust for treatment.

### Young women

In the present study, young age (<40 years) was positively associated with high BCSM following invasive breast cancer. In the second diagnostic period (1971 to 1980) the BCSM of young women decreased and then increased again in the following diagnostic period (1981 to 1991). However, it is difficult to interpret these small changes in BCSM as only 44 women under 40 years were diagnosed in the second period and chance may have caused some of the change.

It has been shown that young women have higher grade tumors, which are consequently more aggressive [8,22,23]. Tumors in young women are also more prone to be hormone receptor negative, which makes them less susceptible to respond well to adjuvant endocrine therapy such as tamoxifen [8,23]. Therefore the medical adjuvant therapy form for young women has instead been chemotherapy (introduced in the late 1970s at this institution) [16]. However, according to the results of this study, the BCSM of young women did not decrease in relation to other age groups in spite of the introduction of chemotherapy. Young women had the second highest BCSM in the diagnostic period 1981 to 1991, following the introduction of chemotherapy.

Several previous studies have also found young women to have a higher BCSM than other age groups [6-10,13,24-29]. However, two studies concluded that there was no difference in survival rates between young and middle-aged women [14,30]. The contradictory results may be due to breast cancer being a heterogeneous disease, with the prognostic factor age only having an effect in certain subgroups of breast cancers, such as the stage factors size, ALNI, and distant metastasis. A few previous studies have stratified for stage to evaluate this, with contradictory results. One study reported worse survival rates for young women in stages I and II [10], one large study for stages I to III [9], and one review from 2008 concluded that a worse outcome for young women is found in all stages (I to IV) [11].

Our analysis, stratified for ALNI, demonstrated that the RR increase of young women was especially apparent in ALNI-negative young women; ALNI-negative women aged under 40 years had a worse prognosis than ALNI-negative women in other age categories. As ALNI status is the most important prognostic factor and women who are diagnosed as ALNI-negative are expected to have a favorable prognosis, this is an important finding. A reason for our result could be that the strong confounding factor ALNI had been stratified for, which may reveal a relatively weak prognostic factor; young age. It can also be because adjuvant treatments, such as radiotherapy, chemotherapy, and endocrine treatment, were given mainly to ALNI-positive women [16]. Consequently, the outcome in ALNI-negative women would reveal the natural course of events, where young women were distinguished as having the very worst five-year prognosis out of all age groups. In line with these findings, a previous study has shown the poor prognosis of young women is only to be found in those not treated with adjuvant therapy [29].

In the sensitivity analysis, there was no large difference in BCSM rate between women under 35 years and 35 to 39 years. However, the number of women diagnosed who were under 35 years was small (53 women), hence the statistical power of the analysis was poor.

### Middle-aged women

Middle-aged women (50 to 69 years) had a worse survival rate than the reference group (40 to 49 years) in the first two diagnostic periods. However, in the last diagnostic period, starting in 1981, women aged 50 to 59 years had a similar survival rate and women aged 60 to 69 years had an even better survival rate, as compared to the reference group. Mammographic screening was introduced at this institution in 1976 in a randomized trial, inviting 50% of women aged 45 to 69 to participate [16]. We did not have information on which of the breast cancer cases were detected by mammographic screening. However, it is possible to hypothesize that this may explain some of the decrease in BCSM over the diagnostic periods, seen in women aged 50 to 59 years and 60 to 69 years in this study, as they would have been diagnosed at an earlier stage and subsequently have had a longer time from diagnosis until potential death from breast cancer (a lead time effect) [31]. On the contrary, the youngest and oldest women may still have experienced a relatively late diagnosis and, hence, a continuing higher BCSM rate. Adjuvant therapy was also introduced in the late 1970s. This may also be part of improved survival rates for middle-aged women due to the connection in time.

### Elderly women

Women aged 70 to 79 years had a high BCSM until the last diagnostic period. It may be that diagnosis and treatment improved also for this age category, but slightly later than for middle-aged women.

The strongest association for all age categories was for the oldest women (≥80 years), who had a worse outcome in all analyses. Previous studies have found contradictory results, with old age being shown to be associated with [5,12,13,32], as well as not associated with [4,14] a high mortality rate. One large study even found elderly women with ALNI-negative tumors to have a favorable outcome [15]. Adjustment for stage at diagnosis made the association slightly weaker in the present study, suggesting the possibility of delayed diagnosis. However, the results still remained statistically significant following adjustment for stage. Furthermore, stratification for ALNI did not have a large effect; therefore stage could not explain the entire difference in BCSM rate.

The survival rate for elderly women improved over the diagnostic periods. A reason for this may be that tumors in elderly women are often hormone receptor positive [15]. Hence a high percentage of this age group responds well to endocrine therapy [33,34]. The endocrine therapy tamoxifen was introduced in 1978 at this institution and could have contributed to this improved survival rate [16].

Although the survival rate improved for elderly women over the diagnostic periods, elderly women continued to have a higher relative risk when compared to other age categories. This could be due to elderly women potentially not receiving treatment according to the guidelines. In this material we unfortunately did not have access to data on adjuvant therapy, but we did have information on type of operation and data on whether or not an axillary dissection was performed. This indeed showed that it was more common that older women had been regarded as unsuitable for surgery and an axillary dissection was also less common in these women. Furthermore, it has been displayed in previous studies that women have not received treatment according to the guidelines, especially regarding adjuvant radiotherapy [12,15,35-38]. Other studies have also shown axillary lymph node dissection to be less frequently performed in the elderly [5,36,38]. Comorbidity can naturally lead to elderly women receiving less aggressive treatment, but studies have shown that old age is an independent risk factor for not receiving appropriate therapy after control for comorbidity [12].

## Conclusions

In this study, young (<40 years) and old (≥80 years) age was positively associated with a high BCSM. For women aged under 40 years, this association was predominantly apparent for those with ALNI-negative breast cancer. An age of 80 years or more was a prognostic factor for high BCSM, independent of stage and diagnostic period.

Breast cancer is rare amongst young women, but it is still the most common malignancy in women under 40 years of age [2]. In this study, mortality in ALNI-negative young women was higher than for ALNI-negative women of other age categories. This motivates further studies evaluating if ALNI-negative young women have a poor prognosis.

In this study, an age of 80 years or more was a prognostic factor for high BCSM, independent of stage and diagnostic period. Approximately one third of breast cancer is diagnosed in women aged 70 years or more, and around 15% of all breast cancer occurs in women who are aged 80 years or more at the time of diagnosis [2]. With average life expectancy rising, breast cancer is likely to become increasingly frequent in elderly women [39]. Elderly women will also live longer from the time of diagnosis and subsequently have an increased risk of metastasis. Hence, it is important to make sure that elderly women also receive treatment according to the guidelines.

## Consent

The current study used information in registers and clinical notes already obtained. No additional contact was taken with previous patients, out of whom many had died since their diagnosis. The Regional ethical committee in Lund, Sweden (approval number: LU-Dnr 615/2004) recommended that previous patients should be informed about the study, and the possibility to withdraw, using advertisments in local newspapers and this was done before the analyses began.

## Abbreviations

ALNI: Axillary lymph node involvement
BCSM: Breast cancer-specific mortality
CI: Confidence Intervals
RR: Relative Risk
